# The Value of lncRNA GHET1 as a Prognostic Factor for Survival of Chinese Cancer Outcome: A Meta-Analysis

**DOI:** 10.1155/2019/5824190

**Published:** 2019-12-01

**Authors:** Junying Song, Xian Chen, Qingwu Tian, Limin Lun, Qing Wang, Hongwei Wang, Xiaopeng Li, Zhongqiang Liu, Liyuan Tian, Xuran Jing, Yunyuan Zhang, Runhua Tian

**Affiliations:** ^1^Department of Biochemistry and Molecular Biology, Medical College of Qingdao University, Qingdao 266021, China; ^2^Department of Clinical Laboratory, The Affiliated Hospital of Qingdao University, Qingdao 266003, China

## Abstract

**Aim:**

There is increasing evidence that high expression levels of the gastric carcinoma highly expressed transcript 1 (GHET1), a long noncoding RNA (lncRNA), are associated with cancer prognosis and may be used as a valuable biomarker for cancer patients. The purpose of this meta-analysis was to analyze existing data to reveal potential clinical applications of GHET1 for cancer prognosis and tumor progression. All of these studies included in this meta-analysis were collected through a variety of retrieval strategies; and the enrolled articles were qualified via the meta-analysis of enrolled studies in epidemiology (MOOSE) and the preferred reporting items for systematic reviews and meta-analyses (PRISMA) checklists.

**Materials and Methods:**

The literature collection was performed by a comprehensive search through electronic databases for studies published on or before March 10, 2019. These included the Cochrane library, PubMed, Embase, Web of Science, Springer, Science Direct, and three Chinese databases: CNKI, Weipu, and Wanfang. Seven studies that met the specified criteria were analyzed in the present research.

**Results:**

The combined results indicate that an elevated GHET1 expression level is significantly associated with poor overall survival (OS) (HR = 2.40, 95% CI: 1.87–3.08, *p* < 0.001) and tumor progression (III/IV vs. I/II: HR = 1.80, 95% CI: 1.48–2.18, *p* < 0.001) in multiple cancers. The elevated GHET1 expression was also associated with lymph node metastasis (LNM) (HR = 2.44, 95% CI: 1.86–3.20, *p* < 0.001) in Chinese cancer patients*. Conclusions.* The present findings indicate that an increased GHET1 expression level is associated with poor OS, tumor progression, and LNM in patients with multiple tumors and may serve as a useful prognostic biomarker in Chinese cancer patients.

## 1. Introduction

Cancer is a major threat to human health over the world, with an ever increasing prevalence rate [1, 2]. Although tremendous improvements in cancer treatment continue to be achieved, the long-term survival rate remains unsatisfactory low for many types of cancer. The molecular mechanisms underlying oncogenesis and tumor progression are still not fully elucidated; and this, in turn, restricts the prognostics for cancer patients. It is therefore urgent to identify new, effective biomarkers for early diagnosis and prognosis and to serve as ideal therapeutic targets for cancer patients.

As a class of endogenous noncoding RNA, long noncoding RNA (lncRNA) has a broad range of molecular and cellular functions, including chromatin modification, gene imprinting, alternative splicing, dosage compensation, nuclear-cytoplasmic trafficking, and inactivation of major tumor suppressor genes [3–5]. There is accumulating evidence of dysregulated lncRNAs in various cancers, and it has been suggested that those greater than 200 nucleotides in length may contribute to cancer development and progression [6, 7]. Repeated findings suggest that lncRNAs may participate in a wide range of biological pathways with underlying oncogenesis and progression [8]. Therefore, lncRNAs have attracted considerable attention as a class of modulators and may serve as potential biomarkers for cancer patients [9–12].

Long noncoding RNA gastric carcinoma highly expressed transcript 1 (lncRNA-GHET1), located in an intergenic region on chromosome 7, is expressed in a broad range of cancer tissues. There is emerging evidence from fundamental and clinical studies which show that lncRNA-GHET1 participates in tumorigenesis and that elevated levels are associated with a poor prognosis in multiple types of cancers. The majority of studies, however, that has reported the prognostic value of GHET1 was limited by a small sample size and controversial results. We, therefore, conducted this quantitative meta-analysis to investigate the prognostic value of GHET1 in various cancers.

## 2. Materials and Methods

### 2.1. Literature Search

Articles published in English that related to the prognostic value of lncRNA GHET1 and tumor progression were eligible for the current meta-analysis. A comprehensive search was conducted in several electronic databases for studies published on or before March 10, 2019. These databases include PubMed, Web of Science, Embase, ISI Web of Knowledge, Cochrane Library, BioMed Central, Springer, and Science Direct, together with three Chinese databases: CNKI, Weipu, and Wanfang. The following keywords for the online search in these databases were included: (“long noncoding RNA-” OR “noncoding RNA-” OR “lnc RNA-” OR “gastric carcinoma highly expressed transcript 1” OR “GHET1”) AND (“carcinoma” OR “cancer” OR “tumor” OR “neoplasm”) AND (“prognosis” OR “prognostic” OR “metastasis” OR “metastatic”). The reference lists of the primary publications were also manually searched to find potential eligible studies.

### 2.2. Inclusion and Exclusion Criteria

We used the following selection criteria to identify eligible studies: (1) a definite diagnosis or histopathology that was confirmed for cancer patients; (2) studies investigating the prognostic features of lncRNA GHET1 in any malignant patients; and (3) enough information for the computation of pooled hazard ratios (HR) and 95% confidence intervals (CIs). Exclusion criteria for the articles included (1) studies absent of prognostic outcomes; (2) duplicated publications; and (3) nonhuman research, correspondences, case reports, letters, review articles, and other studies without original data.

### 2.3. Data Extraction and Quality Assessment

Two authors (JYS and LYT) carefully reviewed the information such as titles, abstracts, full texts, and reference lists of each eligible article independently. The enrolled studies were then qualified by meta-analysis of observational studies in epidemiology (MOOSE) and preferred reporting items for systematic reviews and meta-analyses (PRISMA) checklists (Supplementary Tables [Supplementary-material supplementary-material-1] and [Supplementary-material supplementary-material-1]) [13]. For cases where the eligible literature only provided the data as Kaplan–Meier survival curves, the Enguage Digitizer (version 4.1) software was used to extract the survival information from the graphical plots, based on previous described methods [14–16]. The extracted items were discussed and any contradictions were arbitrated by a third investigator (YYZ) to reach a consensus. Additionally, the necessary elements from the enrolled articles were extracted: first author's name; publication year; cancer resources; tumor type and stage; total cases; follow-up period; lncRNA GHET1 detection method; cut-off values; and HRs and corresponding 95% CIs.

### 2.4. Statistical Analysis

This meta analysis was performed with Stata SE 12.0 (Stata Corporation) and RevMan 5.3 software. The main statistical index, HRs and 95% CIs, was calculated for the aggregated patient survival and tumor progression results. Heterogeneity among studies was determined by *I*^2^ statistics. The fixed effects model was conducted in the studies with no obvious heterogeneity (*I*^2^ < 50%) [16–19]. Potential publication bias was evaluated by performing Begg's bias test. Sensitivity analysis was used to show influence by any individual study. A *p* value of <0.05 was considered statistically significant.

## 3. Results

### 3.1. Eligible Studies

**Figure 1:**
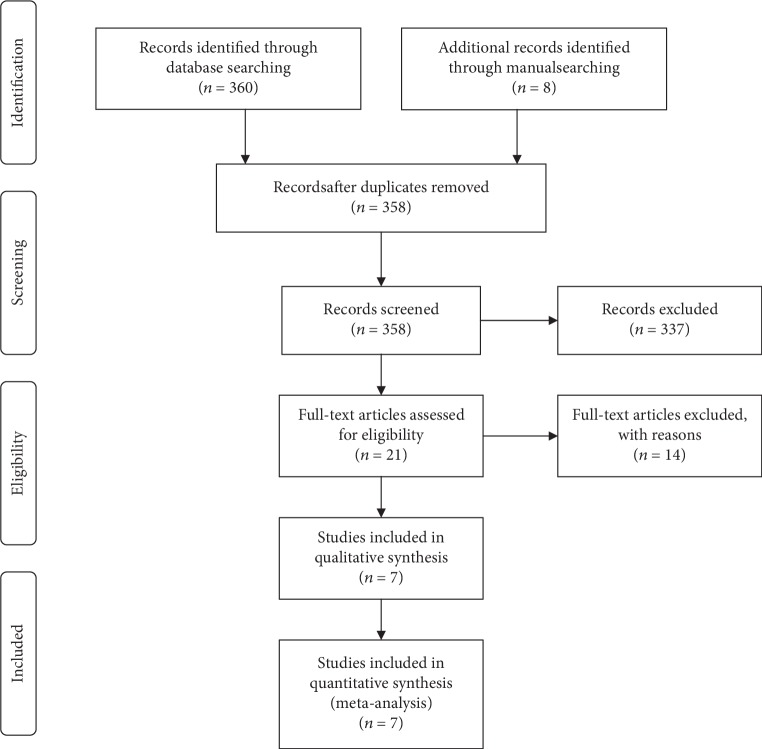
Flow diagram of the study search and selection process.

After the preliminary online search, 368 publications in total were retrieved from the electronic databases. After removing the duplicates, there were 358 potential articles subjected to abstract screens. 337 were excluded because they did not match the inclusion criteria. We then carefully assessed the full texts of the remaining 21 articles; of which another 14 were removed according to the exclusion criteria. Ultimately, seven articles were enrolled in this study [20–26]. The literature screening processes are presented as a flow diagram (Figure 1).

### 3.2. Study Characteristics

The main features of the seven enrolled studies were that they include a total of 464 participants whose relevant data are summarized in Table 1. All of the patients were from China, and qRT-PCR was used to detect lncRNA GHET1 expression levels in these included studies. The cancers evaluated included breast cancer, esophageal squamous cell carcinoma, gastric cancer, head and neck cancer, non-small cell lung cancer, and pancreatic cancer. Notably, the median was selected as the cutoff value in different studies. Six of the seven articles were focused on the association of GHET1 with lymph node metastasis and tumor progression. Five studies investigated the expression level of GHET1 and overall survival (OS) of Chinese cancer patients.

### 3.3. Meta-Analysis

**Figure 2:**
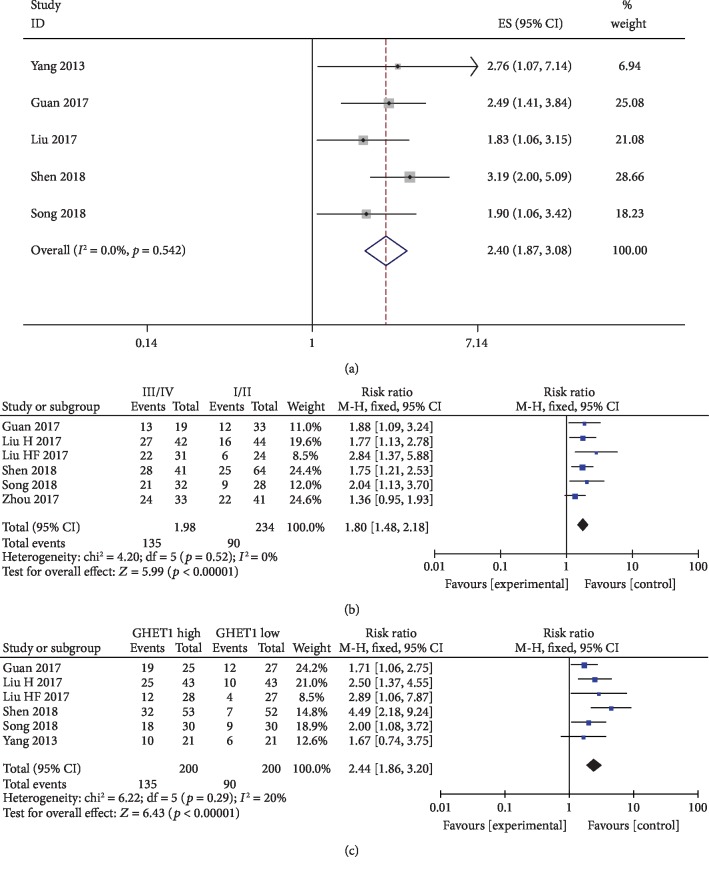
(a) Forest plot for the association between GHET1 expression levels and overall survival (OS). (b) Forest plot for the association between GHET1 expression and TNM stage (III/IV vs. I/II). (c) Forest plot for the association between GHET1 expression and LYM.

Figure 2 presents the forest plot results for lncRNA GHET1 and patient outcomes. A fixed effects model was utilized to calculate the pooled effect size because no significant heterogeneity was observed among the enrolled studies (*I*^2^ = 0%). The combined results indicated that elevated GHET1 expression levels were significantly correlated with poor OS (HR = 2.40, 95% CI: 1.87–3.08, *p* < 0.001) and tumor progression in multiple cancers (III/IV vs. I/II: HR = 1.80, 95% CI: 1.48–2.18, *p* < 0.001) (Figures 2(a) and 2(b)). The elevated GHET1 levels were also associated with lymph node metastases (HR = 2.44, 95% CI: 1.86–3.20, *p* < 0.001) (Figure 2(c)).

### 3.4. Publication Bias

**Figure 3:**
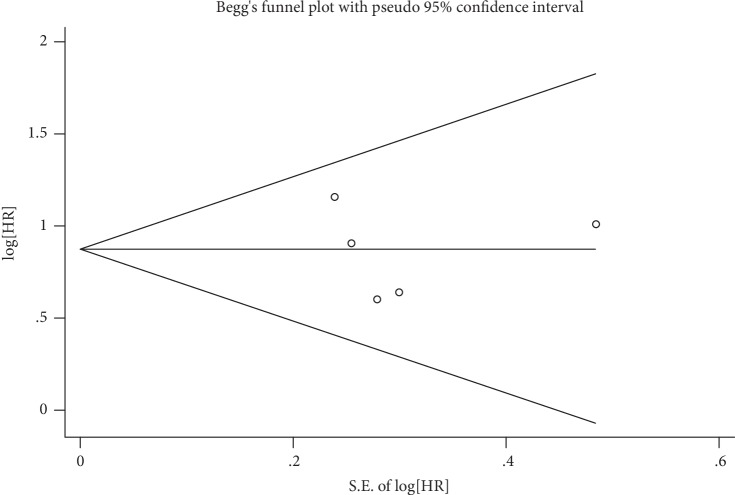
Begg's test of publication bias for overall survival.

To evaluate publication bias in this meta-analysis, the indicated studies were included in a Begg's bias test (Figure 3). The result of Begg's test revealed the absence of significant publication bias (*p* = 0.782).

### 3.5. Sensitivity Analysis

**Figure 4:**
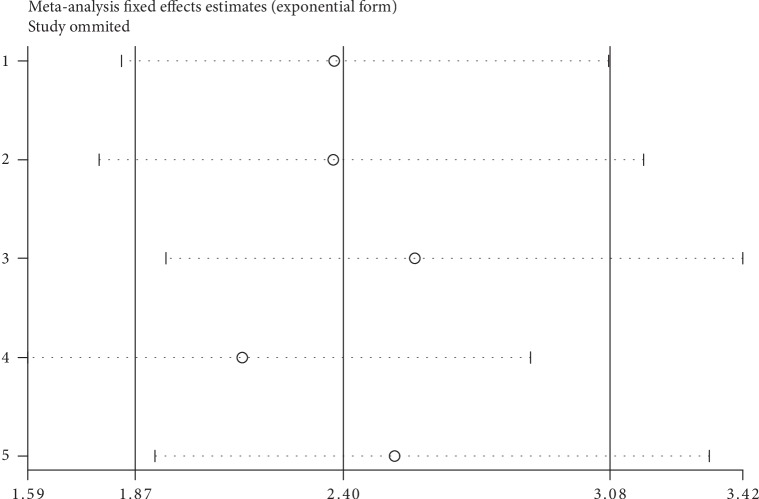
Sensitivity analyses of studies concerning GHET1 and overall survival.

Through sensitivity analysis, it was demonstrated that the pooled GHET1 HR was not significantly affected by the exclusion of any single study (Figure 4).

## 4. Discussion

In the past few decades, lncRNAs had been defined as transcriptional noises, because most of them are produced by intergenic and intron regions of genomes, and lack protein coding ability. In recent years, scientists have made great contributions to the discovery that lncRNAs regulating target gene expression and acting as oncogenes or tumor suppressors. Along with a rapid expansion of high throughput genomic sequencing technologies, lncRNAs have been shown to be useful biomarkers to more precisely evaluate the prognosis of various tumors. There is mounting evidence to suggest that overexpression of lncRNA GHET1 is correlated with poor prognosis and progression in cancer patients. Most studies, however, that reported the prognostic value of GHET1 expression in cancer patients were limited by small sample size. To the best of our knowledge, there have been no previous systematic meta-analyses regarding lncRNA GHET1 expression and cancer patient outcomes.

LncRNA GHET1, a novel lncRNA located in an intergenic region on chromosome 7, has been found to be significantly upregulated in several types of cancers. The molecular mechanisms underlying the oncogenesis and tumor progression are gradually being unveiled. LncRNA GHET1 is activated by histone 3 lysine 27 (H3K27) acetylation and promotes hepatocellular carcinoma (HCC) tumorigenesis through regulating activating transcription factor 1 (ATF1) [27]. LncRNA GHET1 may also promote HCC cell proliferation by silencing Krüppel-like factor 2 (KLF2) [28]. LncRNA GHET1 could promote osteoblast proliferation and differentiation by inhibiting PTEN [29]. Song et al. found that lncRNA GHET1 promoted the cancer progression via EMT in breast cancer [26]. Yang et al. observed that lncRNA GHET1 promoted gastric cancer cell proliferation by increasing c-Myc mRNA stability [20]. GHET1 knockdown significantly decreases the expression of vimentin and N-cadherin in ESCC tissues [22]. Reduced GHET1 expression is related to the inhibition of LATS1/YAP pathway in NSCLC cells [21]. These evidence encouraged us to investigate the relationship between lncRNA GHET1 and cancer prognosis.

Seven published studies that included 464 patients were pooled in this analysis. Several kinds of tumors, including as breast, esophageal squamous cell, gastric, head and neck, non-small cell lung, and pancreatic, were evaluated in the present study. The analyses showed the pooled HR was 2.40 (95% CI: 1.87-3.08, *p* < 0.001) and 1.80 (95% CI: 1.48-2.18, *p* < 0.001) for OS and tumor progression at all cancer types. We also revealed that elevated GHET1 expression was predictive of high risk of LNM (HR = 2.44, 95% CI: 1.86–3.20, *p* < 0.001). Our analysis demonstrated that high expression levels of lncRNA GHET1 are an unfavorable predictor of the clinical outcomes for cancer patients. As analysis was performed in accordance with the recommendations of PRISMA statement, the methodology and results of our research are relatively credible. A fixed effects model was used in most of our analysis which makes our results considerably accurate.

Limitations should be considered that all of the studies enrolled were conducted on Han Chinese; therefore, our results may best elucidate the correlation of lncRNA GHET1 with Asian patients. To strengthen our results, well-designed clinical studies and multiethnic clinical research should be carried out before the application of lncRNA GHET1 as biomarker for global cancer patients' outcomes. Even though inherent deficiencies exist, the present results suggest that promoted lncRNA GHET1 expression levels are associated with OS and lncRNA GHET1 may be used as a prognostic marker for cancer patients.

## 5. Conclusion

Elevated lncRNA GHET1 levels in cancer tissues are significantly related to a greater risk of mortality, progression, and metastasis. Therefore, lncRNA GHET1 could be used as a novel clinical biomarker or therapeutic target for Chinese cancer patients.

## Figures and Tables

**Table 1:** Summary of the seven included studies.

Study	Origin of population	Study design	Disease	*N*	Stage	GHET1 assay	Survival analysis	Metastasis analysis	Hazard ratios	Follow-up (months)
Yang 2013[20]	China	R	GC	42	NA	qRT-PCR	OS	DM/LNM	KM	40
Guan 2017[21]	China	R	NSCLC	52	I/II, III/IV	qRT-PCR	OS	LNM	HR/KM	55
Liu HF 2017[22]	China	R	ESCC	55	I/II, III/IV	qRT-PCR	NA	LNM	NA	NA
Liu H 2017[23]	China	R	HNC	86	I/II, III/IV	qRT-PCR	OS	LNM	KM	70
Zhou 2017[24]	China	R	PC	64	I/II, III/IV	qRT-PCR	NA	DM	NA	NA
Shen 2018[25]	China	R	NSCLC	105	I/II, III/IV	qRT-PCR	OS/PFS	LNM	KM	62
Song 2018[26]	China	R	BC	60	I/II, III/IV	qRT-PCR	OS	LNM	KM	60

DM: distant metastasis; LNM: lymph node metastasis; R: retrospective study; BC: breast cancer; ESCC: esophageal squamous cell carcinoma; GC: gastric cancer; HNC: head and neck cancer; NSCLC: non-small cell lung cancer; PC: pancreatic cancer.

## Data Availability

All data supporting this meta-analysis are from previously reported studies and datasets, which have been cited. The processed data are available from the corresponding author upon request.

## Supplementary Materials

Supplementary Materials Table S1: MOOSE (Meta-analyses Of Observational Studies in Epidemiology) checklist. Table S2: PRISMA (Preferred Reporting Items for Systematic Reviews and Meta-Analyses) checklist.
